# Growing Up with Haemophilia: Quality of Life and School Functioning of a Group of Mexican Adolescents

**DOI:** 10.5334/cie.148

**Published:** 2025-03-21

**Authors:** Maricela Osorio-Guzmán, Carlos Prado-Romero, Santa Parrello

**Affiliations:** 1 Universidad Nacional Autónoma de México, MX; 2 Universitá degli Studi di Napoli Federico II, IT

**Keywords:** Haemophilia, adolescence, academic functioning, quality of life correlated to health

## Abstract

Haemophilia, like many other chronic rare diseases, causes limitations to daily activities, stunting the growth and impairing the quality of life as well as the psychosocial functioning and education of those affected. However, these consequences are distributed differently among those affected depending on the available contextual resources. The aim of this study was to analyse the variables associated with the academic functioning and quality of life of a group of 57 Mexican adolescents ( \[\overline x \] = 14,16 years old; *SD* = 1,91) suffering from haemophilia, in order to identify specific protective factors. Two tools were employed, an ad hoc questionnaire to collect general data and the Paediatric Quality of Life Questionnaire (PedsQL). The participants reported repercussions such as pain (75.4%), having difficulties walking (19.3%) and building relationships with other adolescents (17.5%), missed school days (78.9%), and/or having trouble keeping up with academic activities (38.6%). Adolescents with Type A Haemophilia displayed higher levels on the scales investigating social relations (*t* = 2,356; *p* < 0,05; δ = 1,44), academic functioning (*t* = 3,713; *p* < 0,01; δ = 2,27), psychosocial health (*t* = 2,561; *p* < 0,05; δ = 1,56), and total health-related quality of life (HRQoL) (*t* = 2,467; *p* < 0,05; δ = 1,49) than their peers with Type B. The results indicate that haemophilia has an impact on the adolescents’ global development and their academic performance; however, this impact is reduced by the presence of some of contextual variables/resources.

## Introduction

Chronic health disorders represent a public health problem and have become an economic, political, social, familial, and personal challenge, especially in emerging countries such as Mexico. Data from the United States suggest that between 2009 and 2014, chronic diseases represented more than 60% of the causes for hospitalisation of children and adolescents between 1 and 17 years old (Heslin et al., 2018). Moreover, in these cases hospital admissions may be sudden and frequent (Dunbar et al., 2019). Chronic diseases permeate all areas of a person’s life, family, and environment. As such, they cause important physical changes, as well as psychological, emotional, cognitive, behavioural and relational developmental disorders (Capurso et al., 2024; Osorio-Guzmán et al., 2014; 2015; 2017a). This is especially true in patients affected by paediatric chronic diseases, who are considered a high-risk population since the disease itself can alter growth and development parameters and affect life skills.

The literature on the impact of chronic diseases on students’ academic performance reveals evidence of both direct (educational) and indirect (social and psychological) impacts on education (Thongseiratch & Chandeying, 2020). In terms of direct impact, the severity of the disease and the adverse effects of certain treatment protocols can contribute to cognitive impairment, impacting the developmental process. For example, childhood leukaemia survivors face significant learning difficulties due to brain radiation and aggressive chemotherapy, which affect memory, attention, and processing speed. In other cases, the symptoms of the disease or medication can cause fatigue, drowsiness, and irritability, thereby reducing motivation to study (Martinez et al., 2011). With regard to indirect impacts, children experience frequent hospitalisations (Franchini & Manucci, 2012; Humphries & Kessler, 2013; Muñoz & Palacios, 2015; Osorio-Guzmán et al., 2019), which often contributes to stigmatisation, overprotection, and/or excessive permissiveness, creating difficulties in communication and possibly leading to anxiety and depression (Geerts, et al., 2008; Osorio-Guzmán et al., 2016). They also cause prolonged school absences, which alters the educational process and increases the possibility of academic delays and failures (Di Cola et al., 2023; Hamid et al., 2019; Limperg et al., 2017; Osorio-Guzmán et al., 2017b). However, the effects are never linear: In the case of both physical and neurological chronic diseases, it is believed, for example, that self-concept may moderate the effects of school absenteeism on academic performance (Strothers, 2020).

To address this issue, countries following United Nations guidelines (Organización de las Naciones Unidas [ONU], 2015) have established programs ensuring hospitalized patients’ right to inclusive and equitable education, fostering their personal and social development. For example, in Mexico, the programme “*Sigamos aprendiendo… en el hospital*,” managed by the public health sector, aims to allow students admitted to the hospital to continue the teaching/learning process through a teaching model based on “Basic Education Curriculum and Plan” implemented in cooperation between the educational system and the health sector. The objective is to let students re-enter school upon their discharge from the hospital without any gaps or delays in their education. The programme also includes a training component for teachers to enable them to acquire the necessary teaching skills for specific educational interventions (Gobierno de México, 2017; Instituto Mexicano del Seguro Social [IMSS], 2019). Data from 2018 show that the programme was operating throughout the Mexican Republic, comprising 193 classrooms in 106 public hospitals (Gobierno de México, 2018). To date, there are no specific data on the programme’s scope and results. However, there are data on the educational contribution made by the patients’ families to avoid lags or gaps in their children’s learning. In other words, it is the caregivers who are in charge of linking the home-hospital school system, most of the time creating a support network with teachers, mothers of other patients and mothers of their classmates to achieve educational goals. In this regard, Osorio-Guzmán and Graña (2017) noted that 66% of patients regularly attend school and attend the grade that corresponds to their age, despite parents expressing concern for the future of their children, highlighting school absenteeism as a consequence of inadequate care, and especially a lack of information from health personnel (De la llata, et al., 2021).

### Haemophilia

As mentioned, chronic diseases impact virtually every aspect of the individual, their family, their immediate environment, and the healthcare system; this is especially true with haemophilia diagnoses, since being affected by a chronic disease such as this requires special care, treatments, information, training, and support (Alviz-Amador et al., 2021; Liesner et al., 1996; Manco-Johnson et al., 2007; Osorio-Guzmán et al., 2013; 2017b; 2019; Srivastava et al., 2013), which can lead to feelings of stress, guilt, and frustration in both patients and their families (Barlow et al., 2007; Cassis et al., 2012). A total of 5,547 people have been recorded as having a diagnosis of haemophilia in Mexico (Boletín de la Comisión de Salud, CONCANACO, 2023). In Mexico, haemophilia is on the list of the 20 diseases classified as rare (Secretaría de Salud, 2019) and is defined as an haemorrhagic disease characterised by a qualitative (decreased function of the antigen responsible for coagulation) or quantitative aspect (decreased quantity of the antigen) of factor VIII (haemophilia A) or IX (haemophilia B) of the clotting factors (López-Arroyo et al., 2021; Osorio-Guzmán et al., 2017b). This has grave implications at the biopsychosocial level, as the clinical manifestations of the disease can occur in the following ways:

1. Mild haemophilia (factor level 6–40%): haemorrhages occur following severe injuries or surgery; patients can live without spontaneous bleeding, and articular bleeding is rare;
2. Moderate haemophilia (factor levels 1–5%): haemorrhages happen even following minor injuries, which tend to occur at least once per month; articular bleeding is permanent;
3. Severe haemophilia (factor level below 1%): spontaneous haemorrhages occur between 1 and 2 times per week, accompanied by constant internal bleeding, which can be localised not only in the joints, but also in muscles and even at the level of the central nervous system (Manco-Johnson et al., 2007; Prado & Osorio-Guzmán, 2022; Srivastava et al., 2013).

Thus, the evolution of the disease is characterized by complications due to recurring haemorrhages, which cause physical disability and often put the patient’s life at risk (López-Arroyo et al., 2021; Osorio-Guzmán et al., 2013; Shapiro et al., 2001; Usner et al., 1998). The most frequent haemorrhages are internal and thus not visible; they mostly affect joints such as knees, ankles and elbows, and they cause severely intense pain, constant discomfort and inflammation; and when they are not treated promptly and adequately, they cause grave disabilities, and abnormal growth and development (Alviz-Amador et al., 2021; Osorio-Guzmán et al., 2013; 2017b). Moreover, haemorrhages affecting the central nervous system are life-threatening. This strongly limits one’s activities, causes frequent and sudden hospitalisations or frequent outpatient visits, and requires specific daily care and specialist medical treatment. Haemophilia may also impact neurocognitive development. For example, Hilgartner et al. (1993) found that the disease had effects on cognition at baseline and follow-up, independent of other medical conditions. In children with severe haemophilia, it was also associated with poorer academic achievement, attention, behaviour, emotional and adaptive functioning, and lower IQ and achievement (Loveland et al., 1994; Usner et al., 1998). Attention deficits were also related to central nervous system bleeds (Watkins et al., 2000). Specifically, rates of attention-deficit/hyperactivity disorder (ADHD) and learning disorders were higher than general population rates, 10.3%–29% and 15.8%, respectively (Mayes et al., 1996; Shapiro et al., 2001; Spencer et al., 2009; Wodrich et al., 2003).

Until the 1980s, patients with haemophilia were treated with coagulation factor concentrates derived from human plasma, which lacked viral inactivation and, therefore, were unsafe. More recently, the reduction in viral burden and improvements in the standard of care may have changed the ways in which haemophilia influences cognitive development (Castro et al., 2014). For example, Mrakotsky et al. (2024) conducted a study in United States to assess cognitive, behavioral, and adaptive functions in children and young adults with haemophilia treated according to contemporary standards of care. In terms of neurobehavioral performance, the authors found that most patients with haemophilia had intellectual performance comparable to or higher than that of the general population. Nevertheless, those with attention problems or ADHD, particularly at a young age, showed additional difficulties in executive functioning and adaptive skills. Patients treated with prophylaxis reported fewer problems with emotional adjustment and inattention/hyperactivity than those who received treatment on demand. Socioeconomic factors, such as income level and parental education, were also significantly associated with patients’ neurobehavioral outcomes: Children from backgrounds with lower socioeconomic levels had lower performance in intellectual and executive abilities. These circumstances obviously hinder relations with peers and schoolmates, in turn creating difficulties in overall development and psychosocial functioning, ultimately affecting the quality of school life (Alviz-Amador et al., 2021; Fornari et al., 2024; Limperg et al., 2014; 2017; Mercan et al., 2010; Osorio-Guzmán et al., 2017b; Rambod et al., 2016; Ravens-Sieberer et al., 2005). In particular, suffering from a chronic disease such as haemophilia during adolescence presents great challenges, because in addition to the physical and social changes characterizing this phase, adolescents need to manage themselves and make important decisions concerning the pharmacological treatment – they need to follow a set diet, limit the consumption of substances that can cause addiction, select an adequate type of physical activity – thus adopting a lifestyle that differs from the one led by their healthy peers. All of this can heighten the feelings of injustice and rebellion that are already typical of this age (Martínez-Chamorro et al., 2002; Prado & Osorio-Guzmán 2022). For these reasons, the World Federation of Haemophilia (WFH; 2021) has underlined that it is essential that healthcare systems guarantee access to long-term, regular replacement therapy (prophylaxis) using state-of-the-art therapeutic agents. This way, many negative consequences may be avoided, since treatments help patients affected by severe haemophilia to have healthy joints and thus better levels of quality of life (Ferreira et al., 2018; Manco-Johnson et al., 2007; Osorio-Guzmán et al., 2017c; Srivastava et al., 2013). In addition, differences in context must also be taken into account (Bullinger et al., 2008a; 2008b; Rambod et al., 2018; Ramos-Petersen et al., 2023; Taha & Hassan, 2014; Tantawy et al., 2011). In areas such as Latin America, many families are responsible for care on top of other problems such as poverty, unemployment, and the presence of other family members with chronic diseases. In such cases, a lack of high-quality, complete healthcare services amplifies already existing inequalities (Noser et al., 2016; Osorio-Guzmán & Graña, 2017).

The concept of quality of life correlated with health is a multidimensional construct (Eiser & Morse, 2001; Ferreira et al., 2018; López-Rincón et al., 2019) that is based on three main components: (a) bodily functions/structures, (b) activity, and (c) participation, which can lead to different forms of restriction and limitation (Fernández-López et al., 2010; Remor, 2011). Over time, several tools had been designed to measure quality of life correlated with health. In a recent systematic review of quality of life correlated with the health of children and adolescents affected by Type A haemophilia, Ferreira and colleagues (2018) identified 6 tools used in 29 studies: 2 were specific to haemophilia (Haemo-QoL and CHO-KLAT), and 4 were general (PedsQL, EQ-5D-3 L, KIDSCREEN-52, and KINDL). The present study employed the PedsQL, adapted for the Mexican population by Villaruel-Rivas and Lucio in 2010; it obtained good psychometric characteristics and has been since frequently applied (van den Berg et al., 2015; Varni et al., 2003; Ware & Sherbourne, 1992). According to Boehlen and colleagues (2014), Fischer and colleagues (2016), and van den Berg and colleagues (2015), the quality of life construct correlated with health should always be included in research evaluating therapeutic options for patients suffering from haemophilia, because it is essential for fully understanding the impact of the disease and its treatment on the individual. In the case of children and adolescents, school life is surely the most important part of overall quality of life; nevertheless, there is not an abundance of specific studies centred on its analysis and evaluation.

Paz-Lourido et al. (2020) highlighted the social benefits derived from the inclusion in school of children and adolescents suffering from rare diseases. The problems identified were grouped into seven categories: difficulty with attendance, knowledge about the disease, participation, acceptance, discrimination, safety, and support. Compared to the healthy peers, a child with poor health conditions tends to miss school more often and create fewer social bonds, with negative consequences on social functioning, academic performance, and self-esteem. Another study from the same year (Verger et al., 2020) identified 10 categories that influence a healthy, inclusive, and fair school experience: a clear, early diagnosis, official recognition of the disease, a welcoming school, additional staff, healthy management of school absences, school-hospital coordination (Verger et al., 2021), accommodations in the curriculum, flexibility concerning homework, support to independence, and peer support. Concerning more specifically congenital haemorrhagic diseases, Gigli (2009) studied the teacher’s point of view. Study results underlined the importance of correct information around the disease and of good school-family communication to avert the risk of devaluing prejudices from both the teachers and the classmates. For example, poor information can produce attitudes and behaviours that range from the passivisation and hyper-protection of the student, to the negation of their specific needs. Sommer and Klug (2024) recently described a teacher training programme on the topic of rare disease that improved the teachers’ specific skills and their sense of self-efficacy.

**Figure 1:**
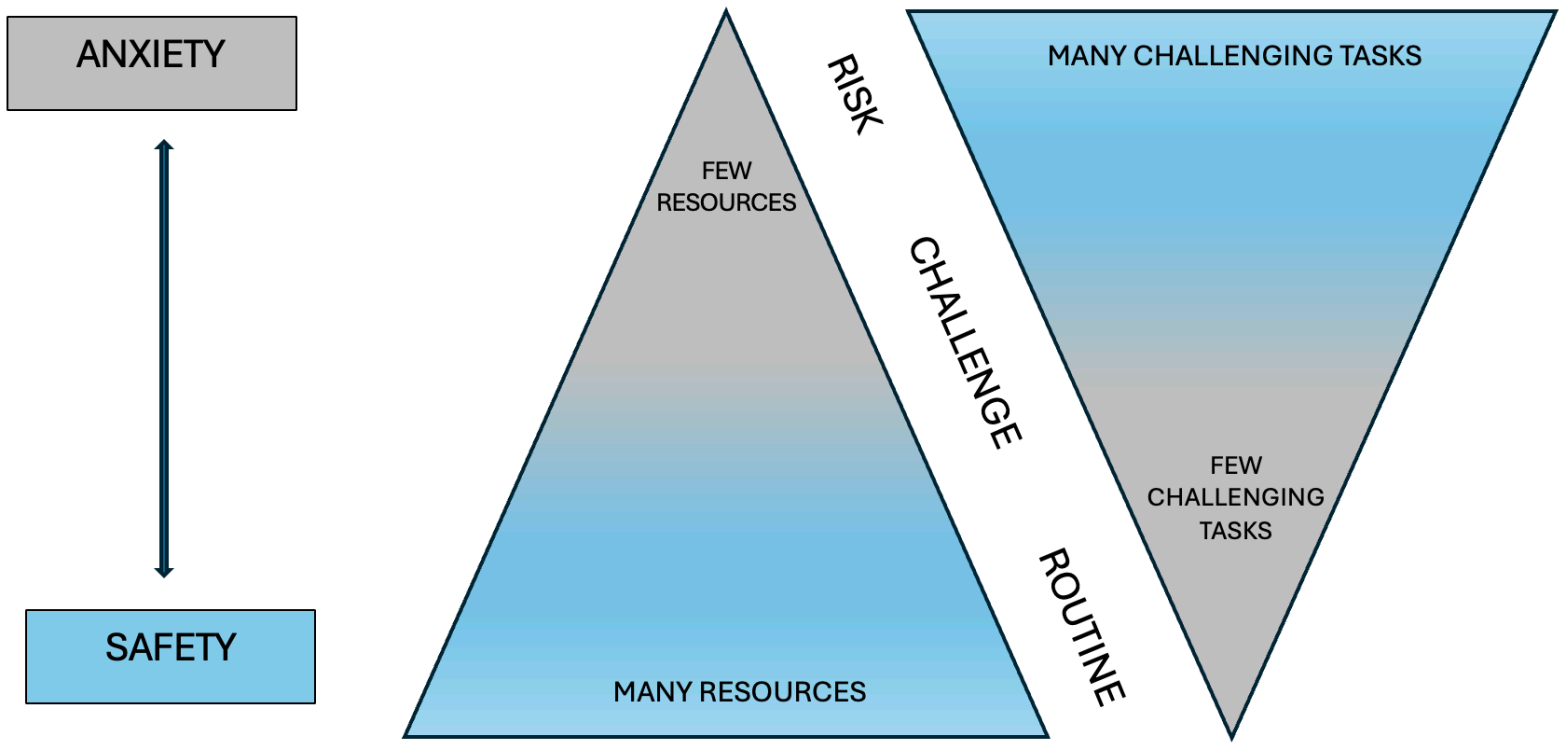
*The Resources/Challenges Model* (Hendry & Kloep, 2002).

In conclusion, the literature underscores the necessity to support children and adolescents with haemophilia or other rare and/or chronic diseases from different angles: outside the school, by family and medical professionals; and within the school, by teachers and peers. In this respect, systemic development theories provide a good theoretical framework. Bronfenbrenner’s (1976) ecologic theory describes human development as a progressive adaptation between the growing individual and the characteristics, more or less mutable, of the “systems” (micro, meso, eso, macro, and chrono) in which they live, such as school. His model stipulates, among other things, that to adequately support development, the various contexts (e.g., family, school, hospital) must be characterized by constructive social interactions, where everyone feels a sense of connectedness and safety, and communicates effectively with each other (Capurso, 2015). Hendry and Kloep (2002) interpreted these context properties in terms of “resources” of the individual (physical, intellective, emotional, social, material, environmental). According to these authors, therefore, development is a string of “challenges;” that is, tasks that require the employment of resources. The challenges can be *normative* (i.e., shared with peers, such as puberty); *quasi-normative* (i.e., shared with peers belonging to the specific cultural group, such as schooling); and *non-normative* (i.e., unique or specific, such as a disease) (Parrello, 2008). If the resources pertaining to the individual context system are adequate, the challenge takes place in a safe context and produces incremental change, thus growth. By contrast, when resources are limited and the number of co-occurring challenges is too high, there is an increased risk of anxiety and stagnation or regression, with cumulative negative effects that affect any subsequent challenge (see Figure 1). The co-occurrence of multiple challenges – such as puberty and schooling alongside the management of a disease – requires a greater mobilisation of resources. It is thus desirable that the resources that are more strictly individual be integrated with or compensated by those of all contexts, from micro to macro.

Against this background, the aim of the present study was to develop an initial picture of the variables associated with the academic functioning and quality of life of Mexican teenagers suffering from haemophilia, in order to identify protective factors, and possibly resources that can be used to orient future health policies.

## Methods

### Participants

Fifty-seven adolescents suffering from haemophilia and registered in the national census of the Haemophilia Federation of the Mexican Republic (FHRM A.C.) participated in the study. A written request to access the target population was submitted to the board of the FHRM A.C. The protocol was reviewed and approved by the Patients’ Association; a cooperation agreement was signed with the directors following all measures and guidelines needed to access the national register of people suffering from haemophilia. All required ethical and privacy criteria were followed. The association was provided with the inclusion criteria of the patients. The register manager sent the list of patients fulfilling these criteria to the person responsible for the present study. The parents of the patients were contacted via e-mail, text messaging, and/or phone call. They were read and/or sent the informed consent form, and the objectives of the study were explained to them. Only once their consent was received did the research begin. Finally, the general project was approved by the Ethical Committee of the UNAM, Facultad de Estudios Superiores Iztacala, Official letter: CE/FESI/122020/1375.

### Instruments

Two tools were employed for data collection.

#### Questionnaire on General Data

This questionnaire, built ad hoc, comprised information about personal data/variables such as age; origin; family structure, family’s socio-economic level, perceived support from family; type of haemophilia, severity of the disease, age of diagnosis, target joints, degree of arthropathy, type of treatment, number of hospitalisations in the past six months, complications in the past year, perceived level of activity, degree of satisfaction in the treatments received by their haematologist; level of education, frequency of sports practice.

#### Questionnaire on Paediatric Quality of Life (PedsQL)

The PedsQL questionnaire was developed by Varni et al. (2001) and validated for the Mexican population by Villarruel-Rivas and Lucio (2010). It has the aim of evaluating the quality of life correlated to the health of paediatric patients, version 13–18 years; it is composed of 23 items divided into four domains: physical, emotional, social, and academic functioning. This tool can be applied to the child and the parent (or guardian) concurrently. Possible answers are recorded on a 5-point Likert-type scale (0 = never any issue, 1 = almost never, 2 = sometimes, 3 = often, 4 = almost always). The internal coherence of the children/adolescents scale is 0.90, and that of the parents’ scale is 0.93.

### Procedure

The research was carried out remotely, using a digital platform for video calls provided by UNAM university.

The collection period spanned from January 2021 to January 2022. On average, the meetings lasted an hour, divided into three parts: 5 minutes of initial exchanges, 45 minutes of questionnaire administration, and 10 minutes of closing remarks, general comments, and goodbyes. During the administration of the instruments, the questions or items were projected on the participants’ screen alongside the possible answer options in order to facilitate the process. Once the data collection was completed, the corresponding database was built for statistical analysis.

### Data Analysis

The sample’s attributive variables were calculated and described, along with the scores obtained on the tools that were answered exclusively by the patients. Students’ *t* statistics and results of an ANOVA were employed to analyse the differences between the subgroups of the sample, as well as Cohen’s *d* and eta-squared to present the entity of these differences. Finally, the strength of the association between variables was assessed using Pearson’s *r*.

## Results

First, the results of the descriptive analysis of the sample’s attributional variables, which emerged from the first questionnaire, will be presented.

The participants’ average age was 14.16 years (*SD* = 1.91). They resided in different states of the Mexican Republic. Concerning their education level, 77.2% studied in the Nivel Básico (primary school, duration 6 years), 21.1% in the Secundaria (secondary school, duration 3 years), and 1.8% in the Nivel Medio Superior (preparatory school, duration 3 years). All participants reported being enrolled in school at present, although at the moment of data collection, due to the COVID-19 pandemic, they were doing remote schooling, in conformity with the programmes enacted by the federal government. Regarding family composition, 70.2% of respondents reported having a nuclear family (mother, father, and siblings), with the number of cohabiting family members ranging between 1 and 10 ( \[\overline x \] = 4.05; *SD* = 1.73; μ = 4). In 87.5% of cases, the main caregiver was the mother; concerning the time dedicated to assisting the patient, 45.6% declared being assisted 24 hours per day, with no breaks ( \[\overline x \] = 14.33; *SD* = 9.53; min = 1 – max = 24) (see Table 1).

**Table 1:** Family Composition and Patient’s Caregiver.


TYPE OF FAMILY	FREQUENCY	PERCENTAGE

Extended	14	24.60

Single-parent	1	1.80

Nuclear	40	70.20

Three generations	2	3.50

**CAREGIVER**	**FREQUENCY**	**PERCENTAGE**

Grandmother	1	1.80

Sister	1	1.80

Mother	50	87.50

Father	3	5.40

Aunt	2	3.60


The families’ socio-economic level was reported as average in 89.5% of cases, and as low in 10.5% of cases. The perception of support from family and friends, and of the quality of healthcare (from one’s own haematologist) was investigated on a scale from 1 to 10. Participants reported high levels of support from their families and the medical equipment, and medium levels of support from their friends (see Table 2).

**Table 2:** Perceived Social Support.


	AVERAGE	STANDARD DEVIATION

Support from family	9.5439	1.01893

Support from friends	7.6842	2.51537

Support from haematologist	9.3333	1.49204


The data on the disease showed that 70.2% of respondents were affected by Type A haemophilia; the rest by Type B; great severity was predominant (42.9%), followed by moderate severity (35.7%), and mild severity (21.4%). The average age of diagnosis was 23.66 months (*SD* = 26.7). Sixty-five percent of the sample were undergoing prophylactic treatment, mostly administered by the main caregivers (66.7%). Regarding complications of haemophilia, 94.7% declared not having any significative bleeding in the past six months; however, 78.9% reported minor bleeding, bruises, or pain in the past year, and 36.8% reported permanent joint damage (ankles 19.0%, knees 28.6%, elbows 52.4%). Moreover, according to what the patients reported, none of them had needed to be hospitalised in the past six months; previous hospitalisations lasted between 2 and 20 days, depending on the severity of the bleeding. None of the interviewees participated in educational programmes within healthcare institutions, none remembered ever having attended any school project during hospitalisation, and none were able to tell whether the hospitals they attended had their own schools. Fifty-four percent reported practicing sports or physical activities, although it was striking that only 46.2% practiced sports recommended for their condition such as swimming or walking; the rest practiced boxing (7.7%) or soccer (15.4%), which was contraindicated.

Concerning the data on quality of life, collected through the PedsQL questionnaire, most participants presented moderate or high levels of risk for most scales (see Table 3). The *academic functioning* area stands out: in this area, half of the sample was at risk (50.9%); some were even at high risk (14.0%). Specifically, 47.4% frequently had attention difficulties, 54.4% had memory problems; besides, many missed school for both medical or hospital examinations (78.9%) and due to the disease’s symptoms (38.6%), a situation that translates into difficulties meeting one’s academic responsibilities (38.6%). The second problematic area was *psychosocial health*, which emerged as at risk for 47.4% of participants. The area of *emotional health* follows, which was at risk for 45.6% of adolescents. *Social relations* also appeared to be problematic: 22.8% of respondents were at risk, and 3.5% at high risk. Specifically, 17.5% of participants showed difficulties in building relationships with other adolescents, 63.2% reported that “doing things” that other “healthy” adolescents can do is impossible for them, and that they have difficulties in keeping physically equal to their peers (43.9%). Concerning the *physical area*, 75.4% declared feeling pain, 56.1% feeling tired and having difficulty doing housework (15.8%), and a considerable percentage had difficulties lifting heavy objects (64.9%), running (29.8%), exercising (45.6%), and/or walking for more than a block (19.3%).

**Table 3:** Percentage of Evaluation of the PedsQL Scales.


SCALES	CLASSIFICATION	%

Physical health	High risk	0

	At risk	14.0

	No risk	86.0

Psychosocial health	High risk	0

	At risk	47.4

	No risk	52.6

Emotional health	High risk	0

	At risk	45.6

	No risk	54.4

Social relations	High risk	3.5

	At risk	22.8

	No risk	73.7

Academic functioning	High risk	14.0

	At risk	50.9

	No risk	35.1

Total HRQoL	High risk	0

	At risk	27.3

	No risk	72.7


The comparative analysis of data began with students’ *t* statistic and Cohen’s *d* to evaluate the existence of significant differences and effect size between the different subgroups of the sample. Not surprisingly, the patients with mild Type A haemophilia had higher scores on the social relations scales (*t* = 2.356; *p* < 0.05; δ = 1.44), the academic functioning scales (*t* = 3.713; *p* < 0.01; δ = 2.27), the psychosocial health scales (*t* = 2.561; *p* < 0.05; δ = 1.56), and total HRQoL (*t* = 2.467; *p* < 0.05; δ = 1.49) compared to peers with Type B haemophilia; however, moderate-severity Type B patients showed higher levels of academic functioning (t = 2.270; *p* < 0.05; δ = 0.55). Regarding the degree of association between the variables, the family’s socioeconomic level was positively associated with academic functioning, which implies that the more the adolescents felt supported by their family, the higher their levels of emotional (*r* = 0.293; *p* < 0.05) and psychosocial health (*r* = 0.291; *p* < 0.05), and the HRQoL (*r* = 0.269; *p* < 0.05). Lastly, there was a positive correlation between the patient’s level of satisfaction with the hematologist’s work and their academic functioning (*r* = 0.288; *p* < 0.05).

## Discussion

Living with a chronic disease has biopsychosocial repercussions that infiltrate all areas of people’s lives, especially in the case of the paediatric population and when the disease is classified as rare. Since chronic and rare diseases have very specific characteristics, they pose particular, non-normative challenges to those who are affected by them; that is, challenges that cannot be immediately shared with healthy peers (Hendry & Kloep, 2002). These challenges add onto the normative challenges typical of this age group, creating the need to employ more resources; for example, to face puberty, the academic experience, and the broadening of social relations. However, the context is not always capable of providing the appropriate resources, which might create unfair consequences on the short and long term (Lum et al., 2017). It is known that haemophilia, without adequate treatment, can affect both growth and developmental parameters, affecting various areas. It is also known that those who grow up with haemophilia often experience hyper-protection and/or excessive permissiveness, stigmatisation, as well as prolonged school absences, which make academic delays and failures more frequent, with consequences including anxiety and depression (Fornari et al., 2024). However, there is still little research on the consequences of haemophilia beyond the physical level (Osorio-Guzmán & Graña, 2017; Osorio-Guzmán et al., 2017a; 2019). To fill this gap in the literature, the present study had the objective of analysing some variables associated with the academic functioning and the quality of life of Mexican adolescents with haemophilia. Below are the most salient findings.

First, the prevalence of adolescents with severe Type A haemophilia stands out in the present study. It is the most common type of haemophilia at a global level (López-Arroyo et al., 2021), but an alarming finding emerges: only a little over half of the sample reported undergoing regular long-term replacement therapy (prophylaxis), as recommended at the international level (Ferreira et al., 2018; López-Arroyo et al., 2021; Osorio-Guzmán et al., 2015; Federación Mundial de Hemofilia [FMH], 2020), while the rest of patients were undergoing different therapeutic regimens resulting in complications, provoking permanent damages to ankles, knees, and elbows (FMH, 2020; Osorio-Guzmán et al., 2015; Rambod et al., 2016). It is important to reflect on the reasons for this situation, which creates inequalities between the young patients, which might be avoided. As mentioned, different authors (Boehlen et al., 2014; Fischer et al., 2016; van den Berg et al., 2015) have noted that it is important to conduct research in the field of paediatric haemophilia, evaluating the quality of life correlated to physical health, in order to understand the impact of this chronic disease and its treatment. However, it is clear that physical health itself suffers from inadequate or insufficient care provided to patients, or from the different compliance that doctors and families manage to obtain from children and adolescents (Liesner et al., 1996; Manco-Johnson et al., 2007; Prado & Osorio-Guzmán, 2022; Srivastava et al., 2013).

Second, school is a fundamental cultural context for development that may reduce the impact of the diversity linked to the limitations caused by physical health issues, offering a place of learning, play, and relationships, fundamental for healthy biopsychosocial growth (Capurso et al., 2024; Yang et al, 2022). Indeed, as Bruner (1996) argued, culture is probably biology’s latest great evolutionary expedient. In Mexico, in order to guarantee the right to inclusion and equity in educational services for hospitalised paediatric patients, different administrations have adopted the UN’s resolutions (2015) and developed official strategies such as the Programme “*Sigamos aprendiendo… en el hospital*” in order to remedy the prolonged school absences and academic delays of hospitalised students (IMSS, 2019). Nevertheless, in the present study no adolescent reported having participated in educational programmes derived from this strategy during their past hospital stays. However, this finding might be due to limitations of the PedsQL as an instrument for detecting school functioning and to the fact that it was not cross-checked with information from parents and teachers. These circumstances make it impossible to generalize this finding and highlight once again the need to evaluate the effectiveness and reach of the programme, on which no specific data have been published at the time of writing this research (Gobierno del Mexico, 2018).

Related to this issue, the school and hospital contexts may not communicate with each other directly and effectively. This has immediate consequences for hospitalized adolescents, as it deprives them of educational continuity and social relationships with their classmates, possibly weakening their sense of belonging to school and class, recognized as an important mediator for the well-being and school functioning of individuals with medical needs (Kirkpatrick, 2020; Tomberli & Ciucci, 2021). In addition, this may also contribute to these adolescents’ perception of the hospital as a place where what matters is only their organic functioning: this does not help them to make constructive sense of their hospital life experience (Capurso et al., 2021). Moreover, the data on academic functioning emerging from the present study seem alarming, given that 14.0% of respondents were at high risk, and 50.9% at risk. The adolescents reported often having trouble paying attention, forgetting things, and missing school (remote classes from the time of the pandemic as well) in order to go to a doctor’s appointment or to the hospital, to pick up their medications, or due to the presence of symptoms of the disease. Thus, they reported having serious difficulties keeping up with their academic assignments. These data are consistent with those of other studies, such as Shapiro and colleagues (2001) and Fornari and colleagues (2024). It is important that teachers learn to manage these matters, relating them to the disease. That is, while it is true that all students should receive help in finding adequate strategies for concentrating and studying, it is also true that the students of concern here should be supported in a specific way, preventing that they not only do not feel discriminated against but also blamed (Gigli, 2009). With regard to school absences, it is not enough that they are “justified” for medical reasons; it is necessary that the school offers adequate remedial measures. The literature has long shown that children with “educational disabilities” (e.g., dyslexia, dyscalculia, intellectual disability) who received significant educational support in school performed better academically than children with chronic illnesses (without educational disabilities) (Martinez & Ercikan, 2009). Fortunately, this was the result of a cultural revolution that pushed schools to be more inclusive of people with disabilities. It is time to create equally excellent educational supports for students with health challenges by implementing adequate preservice training for all new teachers (Irwin et al., 2018).

Third, it is important to monitor the development of adolescents with haemophilia in the various areas connected to the developmental tasks typical of this age. With puberty, the body assumes new importance, and it is not easy to build a physical self starting from the experience of the disease (Bignamini et al., 2012). The ill body imposes limits, which often prolong the experience of depending on others, slowing down the path towards autonomy. Moreover, in many rare diseases, including haemophilia, complications are not immediately “apparent,” which can foster anxiety in the adolescent, who has the difficult task of facing new experiences measuring caution and risk. Finally, the rarity of the condition places the adolescent in a unique situation that is often difficult to communicate and share, with isolation as a possible consequence (Rambod et al., 2016). Moreover, in the present study about half of the participants were at medium or high risk in the areas of psychosocial and emotional health, consistently with data from other recent studies (Buckner, 2018; Fornari et al., 2024; Williams et al., 2016). In particular, some patients perceived receiving medium levels of social support from peers and reported having difficulties in establishing relations with other adolescents; more than half described themselves as not being able “to do things” that other “healthy” adolescents do, and that it is difficult to remain “physically equal” to their peers (Noser et al., 2016; Osorio- Guzmán & Graña, 2017). This is an alarming finding, both because good peer relationships and bonds of friendship favour the process of autonomy building and constitute a protective factor for mental health, and because they have a specific cultural value in the Mexican context (Padilla et al., 2024). It is evident that these adolescents have had very few satisfactory experiences in this area, and this aspect should be better investigated. Naturally, the severity of the disease carries its own weight, but always alongside other contextual factors/resources. For example, patients with mild Type A haemophilia have higher scores in the sections concerning social relations, academic functioning, psychosocial health, and overall quality of life, compared to mild Type B haemophilia patients, as was already highlighted by multiple studies (Fornari et al., 2024; Liesner et al., 1996; Manco-Johnson et al., 2007; Noser et al., 2016; Osorio-Guzmán & Graña, 2017; Shapiro et al., 2001; Srivastava et al., 2013). However, economic resources, a functioning healthcare system that provides cutting-edge medication, and professionals trained to take care of people affected by haemophilia are all essential to help ensure that even patients with more severe conditions can have healthy joints and acceptable quality of life levels (Ferreira et al., 2018).

In the present study, three variables associated with better levels of quality of life were identified: family support perceived by the patient was positively associated with emotional health, psychosocial health, and overall quality of life levels; the family’s socioeconomic level and the level of satisfaction reported by the adolescents regarding the professional performance of their haematologist were both associated with higher levels of academic functioning. For all intents and purposes, these variables can thus be considered protective agents, capable of supporting the growth of adolescents affected by haemophilia. These protective agents, in turn, can be strengthened by suitable health policies that focus on providing help to families and training doctors and teachers.

### Limitations and Future Perspectives

The limitations of this study lie both in the sampling of the participants and the use of a single research tool. However, the data collected encourage a continuation of this line of research, including among the participants and other actors in the life of adolescents with haemophilia, and the use of a wider range of tools, including qualitative ones, and not only self-reports.

## Conclusions

This type of studies on populations affected by specific rare diseases can guide policy decisions, highlighting some priorities. In wealthy countries, quality of life has become the main objective of the therapeutic process; for this reason, it is assumed that healthcare policies must guarantee not only gene therapy with infusion of the missing factor, but also all resources (psychotherapy, physiotherapy, inclusive education, community life, etc.) useful for guaranteeing a life with opportunities not dissimilar from those that a so-called healthy person has (Giordano et al., 2013; Palareti et al., 2020). Ever since 1948, the right to education has been recognised as a fundamental human right, one that is strategic to access other rights such as the right to health, which allow the full development of personality. Indeed, full participation in school life (Anderson et al., 2014) allows students to take care of their own psychosocial health, as well as become more aware of their own rights, including those concerning physical health. However, as discussed above, physical health can have a negative impact on the academic experience if society does not support authentic inclusion, including offering economic, emotional and practical support to the families and promoting good doctor-patient relationships within hospitals. Without such supports and recognition of their rights, growing individuals enter a negative vicious cycle. It is, therefore, of great importance to give visibility to the problems that patients and their families must face daily, and at the same time suggest possible solutions.
